# Formation of Uni-Lamellar Vesicles in Mixtures of DPPC with PEO-b-PCL Amphiphilic Diblock Copolymers

**DOI:** 10.3390/polym13010004

**Published:** 2020-12-22

**Authors:** Aristeidis Papagiannopoulos, Natassa Pippa, Costas Demetzos, Stergios Pispas, Aurel Radulescu

**Affiliations:** 1 Theoretical and Physical Chemistry Institute, National Hellenic Research Foundation, 48 Vassileos Constantinou Avenue, 11635 Athens, Greece; natpippa@pharm.uoa.gr (N.P.); pispas@eie.gr (S.P.); 2 Department of Pharmaceutical Technology, Faculty of Pharmacy, Panepistimioupolis Zografou, National and Kapodistrian University of Athens, 15771 Athens, Greece; demetzos@pharm.uoa.gr; 3 Jülich Centre for Neutron Science JCNS, Forschungszentrum Jülich GmbH, Outstation at Heinz Maier-Leibnitz Zentrum (MLZ), Lichtenbergstraße 1, 85747 Garching, Germany; a.radulescu@fz-juelich.de

**Keywords:** liposomes, vesicles, small angle neutron scattering, Bayesian analysis, block copolymers

## Abstract

The ability of mixtures of 1.2-dipalmitoyl-sn-glycero-3-phosphocholine (DPPC) and the amphiphilic diblock copolymers poly (ethylene oxide)-block-poly(ε-caprolactone) (PEO-b-PCL) to stabilize uni-lamellar nano-vesicles is reported. Small angle neutron scattering (SANS) is used to define their size distribution and bilayer structure and resolve the copresence of aggregates and clusters in solution. The vesicles have a broad size distribution which is compatible with bilayer membranes of relatively low bending stiffness. Their mean diameter increases moderately with temperature and their number density and mass is higher in the case of the diblock copolymer with the larger hydrophobic block. Bayesian analysis is performed in order to justify the use of the particular SANS fitting model and confirm the reliability of the extracted parameters. This study shows that amphiphilic block copolymers can be effectively used to prepare mixed lipid-block copolymer vesicles with controlled lamellarity and a significant potential as nanocarriers for drug delivery.

## 1. Introduction

Polymer-grafted liposomes find numerous applications in different fields of drug delivery and biomedicine [1,2]. They can ameliorate the circulation time of active pharmaceutical ingredients (APIs) in the human blood by avoiding the plasma protein absorption and targeting into the tumor cells [1]. Thermo-activatable polymer-grafted liposomes for low-invasive image-guided chemotherapy have been already developed with minimal adverse effects and rapid interactive therapy [3]. Several routes of administration can be achieved by use of polymer-grafted liposomal delivery platforms, such as the transdermal route which generally exhibit several limitations and pulmonary delivery [4,5]. Some of polymer-grafted liposomes can be characterized as “stealth” due to their ability to avoid the immune system and extend several times the blood-circulation duration by improving the pharmacokinetic prolife of the encapsulated API [6,7]. The PEGylated liposomes be long to that second generation of liposomes and are marked medicines for cancer chemotherapy, too. The mechanism of the protein absorption/aggregation and the parameters that play a key role for these interactions are presented in the recent literature [8,9].

Poly(-caprolactone) (PCL) is a biodegradable and biocompatible polymer with several applications in drug delivery and tissue engineering [10]. Many types of different nanoparticles such as microspheres, scaffolds, films, fibers and micelles have been produced by using this polymer [10]. Poly(ethylene glycol)-ε-poly(caprolactone) has been also used for the preparation of nanocarriers and the delivery of anticancer drugs [11,12]. PEO-b-PLC grafted liposomes were designed and developed, exhibiting controlled-release properties of the encapsulated drug molecule and limited interactions with the plasma proteins [13]. The same results, especially low immunogenicity, were obtained from PEO-b-PCL grafted niosomes [14].

Preparation methods such as extrusion, sonication and freeze thaw produce liposomes at the scale up to 100 nm however in many cases uni-lamellar vesicles coexist with oligo- and multi-lamellar vesicles [15,16,17]. However, production of vesicles with a single lamellarity, uni-lamellar in particular, plays a key role for controlled drug delivery. The studies on model membranes [18] and investigating heterogeneities on vesicular bilayers [19,20] are also important for the design and development of liposomal systems for delivery purposes.

Experimental determination of lamellarity and size in liposomes is crucial [15] and SANS is a powerful method to resolve these issues [21] i.e., to provide details at the nanoscale regarding size distribution and bilayer structure in liposomal formulations [16] and lateral heterogeneities in vesicular membranes [19]. Small angle neutron and X-ray scattering have resolved the morphology, response to stimuli and kinetics of transformation in pharmaceutical liposomes [22].

In a continuation of our efforts to study in depth the properties of polymer-grafted liposomes (characterized as “chimeric” in our previous publications) with different techniques [13] we present a SANS study of mixtures of DPPC phospholipids and PEO-b-PCL block copolymers. This system creates uni-lamellar vesicles of a broad size distribution and aggregates in aqueous solutions. The detailed SANS study proves that the vesicles are stable upon temperature changes and a Bayesian analysis on the SANS profiles prove the subtle thermoresponsive character of the system. Arguments that are based on the mass concentration and size distribution of the vesicles confirm that the block copolymer with the larger hydrophobic block adheres more strongly to the DPPC vesicles. This work demonstrates that standard preparation protocols of liposomes based on amphiphilic block copolymers may lead to the formation of mixed uni-lamellar vesicles.

## 2. Materials and Methods

### 2.1. Materials

The phospholipid 1.2-dipalmitoyl-sn-glycero-3-phosphocholine (DPPC) was purchased from Avanti Polar Lipids Inc. (Alabaster, AL, USA) and was used without further purification. Chloroform of analytical grade was purchased from Sigma-Aldrich Chemical Co and deuterium oxide (D_2_O) 99.90% was purchased from Euriso-top. The diblock copolymers poly(ethylene oxide)-block-poly(ε-caprolactone) (PEO-b-PCL) were synthesized via ring opening polymerization using PEO as the macroinitiator as described extensively elsewhere [23].

Two PEO-b-PCL diblock copolymers were used in this study termed PEO-b-PCL1 and PEO-b-PCL2 corresponding to monomer numbers N_PEO_ = 113 and N_PCL_ = 49 and N_PEO_ = 113 and N_PCL_ = 20 respectively. PEO-b-PCL1 and PEO-b-PCL2 have molar masses of 10,600 and 7700 g mol^−1^, polydispersity (M_w_/M_n_) 1.43 and 1.18 and hydrophobic content (PCL) 53 and 30 % wt. respectively. In the mixtures of the polymers with DPPC, the DPPC/block copolymer molar ratio was 9/1.

### 2.2. Sample Preparation

The thin film hydration method was used for the preparation of mixed diblock copolymer/vesicle systems as in previous studies [13]. The concentration of DPPC in the final solutions was 30 mg mL^−1^, while dilution of stock solutions by the proper amount of water was performed for lower concentrations (10 and 3 mg mL^−1^). Samples were prepared in D_2_O unless otherwise stated. A Julabo thermostat was used to control sample temperature (accuracy 0.01 °C). A time period longer than half an hour at the desired temperature was allowed for equilibration. The temperatures 25, 37 and 45 °C were selected for the experiments. This way there was one temperature below (25 °C) and one above (45 °C) the gel-to-liquid crystalline phase transition [24,25,26,27] of DPPC tail-region bilayers, expected at 41–42 °C. The temperature 37 °C was chosen as it is the physiologically relevant body temperature.

### 2.3. Small Angle Neutron Scattering Experiments

Measurements were performed with the high intensity/wide-q small angle neutron scattering diffractometer KWS-2 at the FRMII reactor (Jülich Centre for Neutron Science). Scattering wave vector q from 0.0014 to 0.51 Å^−1^ was covered by three separate detection configurations (2, 8 and 20 m detection length) and neutron wavelength λ = 4.5 Å. The two-dimensional isotropic scattering data was treated by standard correction and reduction procedures and azimuthally integrated into 1-D scattered intensity I(q). The flat background in the scattering profiles was subtracted from the experimental data. The instrumental resolution function [28,29] Δq(q) was introduced by a Gaussian function and was convoluted [30] with the theoretical SANS profiles $I^{th}\left(q\right)$ i.e., $I^{conv}\left(q\right)\;=\;\frac{1}{\sqrt{2\pi }\Delta q\left(q\right)}\int_{-\infty }^{+\infty }dq^{\prime }\cdot exp\left(-{\left(\frac{q^{\prime }-q}{\sqrt{2}\Delta q\left(q\right)}\right)}^{2}\right)\cdot I^{th}\left(q^{\prime }\right)$. A Schultz distribution of vesicular internal radii was employed to treat polydispersity [31] as $I^{poly}\left(q;R\right)\;=\;\frac{{\left(\frac{z+1}{R}\right)}^{z+1}}{\Gamma \left(z+1\right)}\int_{0}^{+\infty }dr\cdot r^{z}\cdot exp\left(-\frac{z+1}{R}\cdot r\right)\cdot I^{conv}\left(q;r\right)$ where R is the mean internal radius (Results and Discussion). The polydispersity index is defined as $PDI\;=\;{\left(z+1\right)}^{-1/2}$ with as $PDI\;=\;\frac{\sigma_{R}}{R}$ with $\sigma_{R}$ the root-mean-square deviation from the mean radius. In the fitting procedure $I^{poly}\left(q\right)$ was calculated in iterations and the sum of the weighted square differences $\chi^{2}\;=\;\overset{N}{\underset{i\;=\;1}{\sum }}{\left(\frac{I^{poly}\left(q_{i}\right)-I^{exp}\left(q_{i}\right)}{\delta I^{exp}\left(q_{i}\right)}\right)}^{2}$ between N theoretical and experimental intensities was minimized. Experimentally obtained intensities and their uncertainty are $I^{exp}\left(q_{i}\right)$ and $\delta I^{exp}\left(q_{i}\right)$ respectively. A custom made MATLAB code was used for $I^{poly}\left(q\right)$ calculations and for the application of a minimization algorithm based on a Monte Carlo simulated annealing scheme [32]. In the Results and Discussion section the fitting functions $I^{poly}\left(q\right)$ are referred to as I(q).

We implemented the Markov Chain Monte Carlo (MCMC) algorithm proposed by Goodman and Weare [33] to estimate the posterior distributions of the fitted parameters. In this Bayesian inference [34] 100 Markov chains of 1000 steps each were followed after the simulated annealing process had converged (burn-in period). Corner plots [35] were used in order to present any dependence between optimization parameters $P_{i}$ The inter-parameter correlation coefficients were calculated by $r\left(P_{i},\;P_{j}\right)\;=\;\frac{1}{M-1}\overset{M}{\underset{k\;=\;1}{\sum }}\left(\frac{P_{i}^{k}-\langle P_{i}\rangle }{\delta P_{i}}\right)\left(\frac{P_{j}^{k}-\langle P_{j}\rangle }{\delta P_{j}}\right)$ where $\left\langle P_{i}\right\rangle$ and $\delta P_{i}$ are the mean and standard deviation of $P_{i}$ respectively over the space of M pairs of parameters instances $P_{i}^{k}$ and $P_{j}^{k}$.

## 3. Results and Discussion

**Figure 1:**
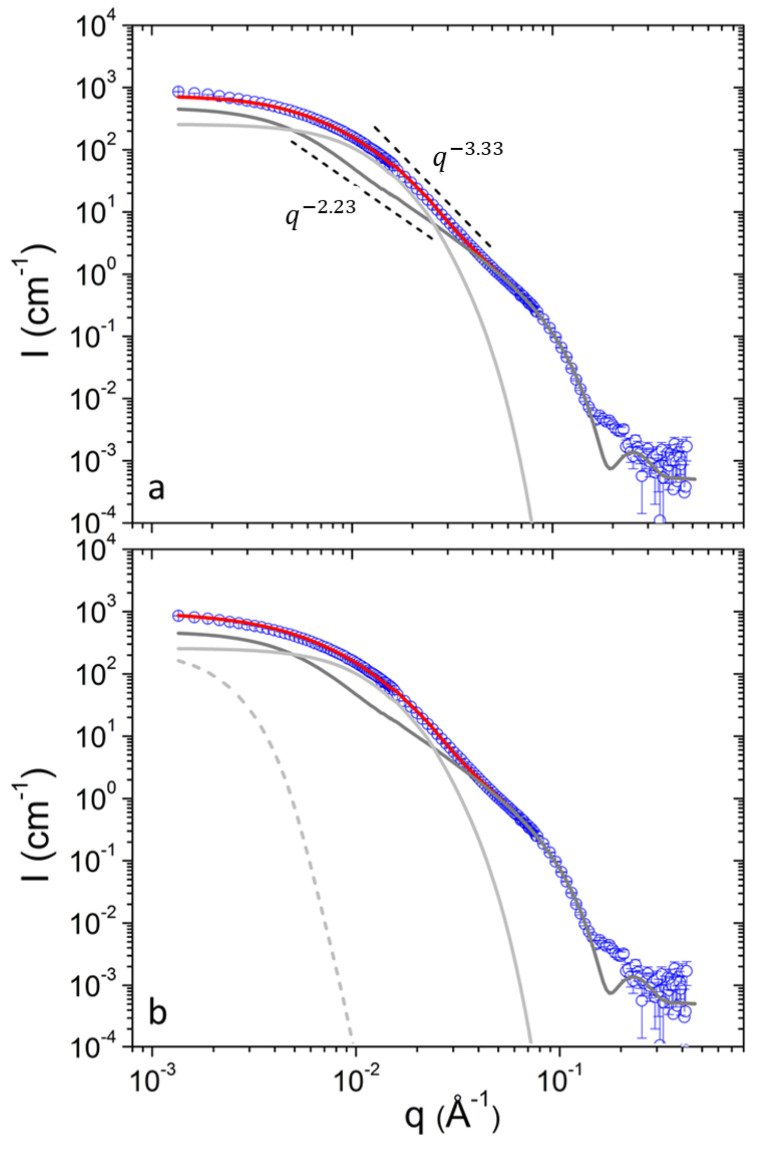
(**a**) SANS profiles from DPPC/PEO-b-PCL1 at 30 (black) mg mL^−1^ at 25 °C. Red line is the best fit with equation 1. Light gray and dark gray lines correspond to contributions from aggregates $I_{agg}$ and vesicles $I_{ves}$ respectively. Characteristic power-law trends are indicated by dashed lines. (**b**) Fitting the data of (**a**) using the additional term $I_{clust}$ (dashed line).

SANS can resolve spatial correlations at length scales 1–100 nm and probes average morphology over macroscopic sample volumes noninvasively. In previous work following the same preparation protocol pure DPPC formed uni- and bi-lamellar vesicles in aqueous solutions with external radii in the order of 25 and 120 nm respectively [17]. In that case a power-law of $I\left(q\right)\sim q^{-2.6}$ was followed at intermediate *q* up to 0.1 × Å^−1^. It has to be noted that scattering from planar interfaces [36] follows $I\left(q\right)\sim q^{-2}$. In the case of DPPC/PEO-b-PCL1 (Figure 1) the situation is completely different. The power-law of $I\left(q\right)\sim q^{-3.33}$ cannot be followed by using a combination of uni- and multi-lamellar vesicles as in previous study [36]. Uni-lamellar vesicles introduce a trend $I\left(q\right)\sim q^{-2.23}$. In addition, multi-lamellar form factors introduce pronounced oscillations that are evidently not present in the data of DPPC/PEO-b-PCL1. In this case the form factor of vesicles had to be combined with the form factor of objects of different morphology. As it is demonstrated in Figure 1a the form factor of uni-lamellar vesicles is adequate to fit the data for $q>3\times 10^{-2}{Å}^{-1}$. In order to fit the data at lower q the superposition with scattering from aggregates and clusters is employed. The presence of aggregates and clusters possibly corresponds to self-associations of PEO-b-PCL copolymers, associations of the copolymers with DPPC phospholipids in a random manner or combination of the two effects. Additionally, it may contain contributions of vesicle-vesicle associations.

The scattering function that was used to fit the data of this work is defined in Equation (1).

(1) $$I\left(q\right)\;=\;I_{ves}\left(q\right)+I_{agg}\left(q\right)+I_{clust}\left(q\right)$$

The scattering amplitude of spherically symmetric objects [37,38] is written as $A\left(q\right)\;=\;\int_{0}^{\infty }4\pi r^{2}\frac{sinqr}{qr}\Delta \rho \left(r\right)dr$. Δρ(r) is the azimuthally averaged radial profile of the neutron scattering length density (SLD) contrast between the particle and the solvent. Scattering from spherical uni-lamellar vesicles (Equation (2)) is defined by the number concentration of vesicles ($N_{ves}$) and the scattering amplitude ($A_{bilayer}\left(q\right)$) which is a superposition of the scattering amplitudes of three separate shells (Equation (3)).

(2) $$I_{ves}\left(q\right)\;=\;N_{ves}\cdot {\left|A_{bilayer}\left(q\right)\right|}^{2}$$

(3) $$\begin{array}{cc} A_{bilayer}(q,B_{out}, & B_{in},R,d_{out},d_{in})\\ & =\;A_{shell}\left(q,B_{out},R,R+d_{out}\right)\\ & +A_{shell}\left(q,B_{in},R+d_{out},R+d_{out}+d_{in}\right)\\ & +A_{shell}\left(q,B_{out},R+d_{out}+d_{in},R+d_{out}+d_{in}+d_{in}\right) \end{array}$$

**Scheme 1:**
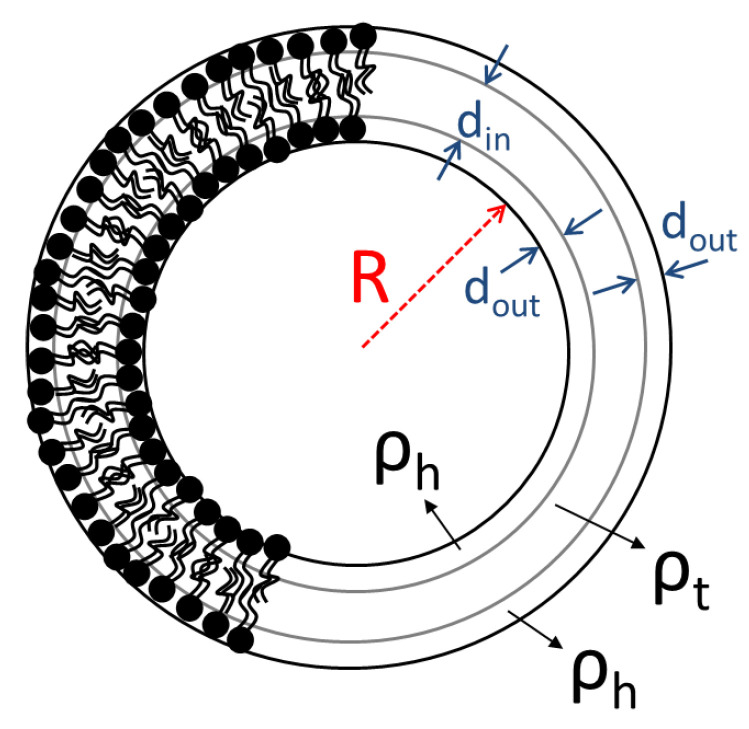
Illustration of DPPC liposomes and main parameters for SANS modelling.

The parameters R, $d_{out}$ and $d_{in}$ are the internal radius of the vesicle, the thickness of the two external layers (head groups) and the thickness of the inner layer (tail region) of the bilayer respectively (Scheme 1). The scattering contrast factors of the separate shells are $B_{out}\;=\;\rho_{h}-\rho_{w}$ and $B_{in}\;=\;\rho_{t}-\rho_{w}$ with $\rho_{w}$, $\rho_{h}$ and $\rho_{t}$ the neutron SLD of the solvent, the hydrated layer and the tail region respectively. Solvent’s SLD $\rho_{w}$ is the volume-average of the SLDs of D_2_O ($6.4\cdot 10^{-6}{Å}^{-2}$) and H_2_O (−0.56$\cdot 10^{-6}{Å}^{-2}$). The scattering amplitude of a single shell is $A_{shell}\left(q,B,R,R^{\prime }\right)\;=\;\frac{4\pi B}{q^{3}}\cdot \left(\left(sinqR^{\prime }-qR^{\prime }cosqR^{\prime }\right)-\left(sinqR-qRcosqR\right)\right)$. It has to be noted that the outer shells are identical in terms of thickness and SLD.

(4) $$I_{agg}\left(q\right)\;=\;G_{agg}\cdot exp\left(-\frac{1}{3}q^{2}R_{g,agg}^{2}\right)+B_{agg}\cdot q^{-D_{agg}}\cdot {\left[erf\left(\frac{q\cdot R_{g,agg}}{\sqrt{6}}\right)\right]}^{3D_{agg}}\cdot exp\left(-\frac{1}{3}q^{2}R_{cut,agg}^{2}\right)$$

The contribution of aggregates to the SANS profiles was modelled by the term of Equation (4). It is the Beaucage model [39] and is a combination of a Guinier term $G_{agg}\cdot exp\left(-\frac{1}{3}q^{2}R_{g,agg}^{2}\right)$ and a power-law term $B_{agg}\cdot q^{-D_{agg}}$. The Guinier term is dominant at $q\cdot R_{g,agg}\ll 1$ whereas the power-law is dominant at $q\cdot R_{g,agg}\gg 1$ (diminished at $q\cdot R_{g,agg}\ll 1$ by the error function). This way, the forward scattering $G_{agg}$ and the radius of gyration $R_{g,agg}$ of an object and the characteristic fractal exponent $D_{agg}$ of its internal structure can be extracted. The factor $B_{agg}$ is used to bridge the two asymptotic terms smoothly [40] i.e., $B_{agg\;}=\;\frac{G_{agg}}{R_{g,agg}^{D_{agg}}}\cdot {\left(\frac{6\cdot D_{agg}^{2}}{\left(2+D_{agg}\right)\cdot \left(2+2D_{agg}\right)}\right)}^{\frac{D_{agg}}{2}}\cdot \Gamma \left(\frac{D_{agg}}{2}\right)$. The exponential cut-off terms $exp\left(-\frac{1}{3}q^{2}R_{cut,agg}^{2}\right)$ fulfills the restriction [39] that scattering from a structural level, in this case aggregates, does not contribute significantly within the q-range that is governed by the correlations dictated by the next-in-sequence hierarchical level i.e., vesicles. In the calculations this is achieved by defining $R_{cut,agg}$ not as a completely free fitting parameter but rather as $R_{cut,agg}\approx R_{g,ves}$.

(5) $$I_{clust}\left(q\right)\;=\;G_{clust}\cdot exp\left(-\frac{1}{3}q^{2}R_{g,clust}^{2}\right)$$

The term $I_{clust}\left(q\right)$ is used in order to capture the low-q upturn in the SANS profiles (Figure 1b). This contribution is more significant at lower concentrations for both copolymers as it will be discussed in the following. However, a single Guinier term is enough to fit the low-q data as this contribution is restricted within a limited q-range. From this the forward scattering $G_{clust}$ and the radius of gyration $R_{g,clust}$ are in principle extracted (Equation (5)). However as the q-range does not allow reliable simultaneous extraction of $G_{clust}$ and $R_{g,clust}$ only the contribution at the lowest q is reported which is defined as $I_{0,clust}\;=\;I_{clust}\left(q_{min}\right)$.

In our previous work [17] DPPC liposomes appeared in combination of uni- and bi-lamellar vesicles. Addition of amphiphilic poly(2-oxazoline) gradient copolymers (MPOx) led to even higher lamellarity which was also temperature dependent. Here only uni-lamellar vesicles appear. Coexistence of uni- and bi-lamellar layers observed by SANS has been also reported on DPPC vesicles prepared by thin film hydration methods [16]. Addition of PEO-b-PCL stabilizes uni-lamellar state which is also stable against temperature increase. It has to be noted that the prepared DPPC/PEO-b-PCL vesicles were stable for at least three weeks, as supported by light scattering experiments (not shown).

The uni-lamellar state can be stabilized against formation of vesicles of higher lamellarity (bi-, tri-, multi-lamellar) by thermal fluctuations that act as an effective repulsive inter-bilayer potential or by the existence of a particular energetically favorable spontaneous curvature [41]. In the case of DPPC/PEO-b-PCL vesicles the absence of vesicles of higher lamellarity upon inclusion of the block copolymer shows that an enhancement of repulsion between possible bi-layers destabilizes any additional bilayers that were present in the pure DPPC vesicles. It can be assumed that the block copolymer chains are incorporated in the vesicles by their hydrophobic block in the hydrophobic tail region, with their hydrophilic block daggling away from the bilayer. This hydrophilic polymer layer effectively enhances inter-bilayer repulsions. A similar effect has been observed in DPPC/MPOx vesicles when the MPOx gradient copolymer was of high hydrophilicity [17]. The effect is opposite than the one observed in DPPC/MPOx vesicles where the presence of gradient amphiphilic copolymers of high hydrophobic content induced formation of multi-layers and the one in hydrophobically modified chitosan which was considered to create attraction between bilayers in unil-lamellar vesicles of the mixed cationic surfactant cetyl trimethylammonium tosylate (CTAT) and the anionic surfactant sodium dodecyl benzene sulfonate (SDBS) leading to bi- and multi-lamellar vesicles [42].

The values $\rho_{t}\;=\;2.70\;\times 10^{-6}{\;Å}^{-2}$ and $\rho_{h}\;=\;-0.31\;\times 10^{-6}{\;Å}^{-2}$ were the optimal fitted values of the SLDs for the tail and head regions respectively and are in agreement with previous works on DPPC vesicles in solution [17,43] and DPPC membranes on the solid water interface [24,44]. These values were adequate in the fits of all data sets in this work for all temperatures and concentrations of PEO-b-PCL1 and PEO-b-PCL2 liposomes. The thickness of the internal layer of lipid tails has been reported to be about 2.8–3.5 nm at room temperature [24,25,43,45]. In our case the resulting thickness of the tail region is $d_{in}\;=\;2.8-3.3\;\mathrm{nm}$ (Table 1). The size of the head groups region has been reported at 0.9–1.1 nm and in our case is $d_{out}≅0.6\;\mathrm{nm}$. We have observed this discrepancy previously [17] and we believe it may be related to the fact that our fitting model does not assume partial overlapping of the separate layers that is normally taken into account in gradually varying scattering length density profiles. As in our recent study, SANS intensity is very weak at *q* > 0.2−0.3 × Å^−1^ and with high uncertainty. Therefore, the SLD profile cannot be resolved to the finest detail at the near sub-nm scale. This way the presence of the copolymer within the DPPC bilayers is not measurable. This is accompanied by the fact that the SLD of the copolymer blocks is between the values of the inner and outer layers of the bilayers, which would not lead to significant changes in the scattering contrast.

The SLD contrast profiles of the vesicles can be confirmed to be extracted in absolute scale by solvent contrast variation experiments (Supplementary Material). It was proved that the fitted SANS profiles could be reproduced by only changing the value of the solvent SLD. This fact also supports that there is no measurable contribution from lateral heterogeneities in the formed membranes. In case they existed, their relative intensity would depend on solvent contrast and SANS profiles would not be reproduced [46]. Contrast variation allows the estimation of the volume-average SLD of aggregates and clusters i.e., $\rho_{agg}\;=\;\rho_{clust}\;=\;1.32\;\times 10^{-6}{\;Å}^{-2}$ (Supplementary Material). Apparently, these objects may consist of PEO-b-PCL and DPPC as the estimated SLD is within the values that correspond to these components.

The internal characteristics of the liposomal bilayers i.e., the thicknesses *d_out_* and *d_in_* do not seem to change as a function of temperature or concentration within experimental uncertainty (see following discussion on parameter uncertainties) in both PEO-b-PCL1 and PEO-b-PCL2 containing liposomes. In the case of pure DPPC a systematic decrease of the internal thickness was found and attributed to the gel-to-liquid crystalline phase transition of the lipid tail region [17,47].

In pure DPPC two different species of vesicles have appeared under the same preparation protocol as the one used here [17]. One population of uni-lamellar vesicles and another one of bi-lamellar vesicles with internal radii of about 20 and 100 nm respectively were found. Their internal characteristics were similar to the ones with added PEO-b-PCL. The size of the uni-lamellar vesicles was unchanged in contradiction to the one of the bi-lamellar vesicles that decreased as temperature increased. However, in that case only weak aggregation of vesicles was reported.

The mean diameter of the vesicles appears to increase as a function of temperature from about 25 to about 30 nm for both polymers (Table 1). This increase is also found in the internal radius of the liposomes. The internal radii R are near 15 nm for both DPPC/PEO-b-PCL1 and DPPC/PEO-b-PCL2 which is smaller in comparison to uni-lamellar DPPC vesicles (~20 nm) and larger in comparison to DPPC/MPOx uni-lamellar vesicles (~10 nm) at room temperature [17]. In uni-lamellar vesicles of the mixed cationic surfactant CTAT and the anionic surfactant SDBS addition of small amounts of hydrophobically modified chitosan led to a significant decrease of the vesicular radius. It was concluded that the presence of the polysaccharide enhanced the bending rigidity of the bilayer leading to higher curvatures [42]. The opposite effect observed here may be related to the incorporation of polymer chains within the bilayer and increase of the surface area of the vesicles. Possibly, the increase in temperature induces stronger hydrophobic interaction between PCL blocks and the hydrophobic interior of the bilayers.

**Figure 2:**
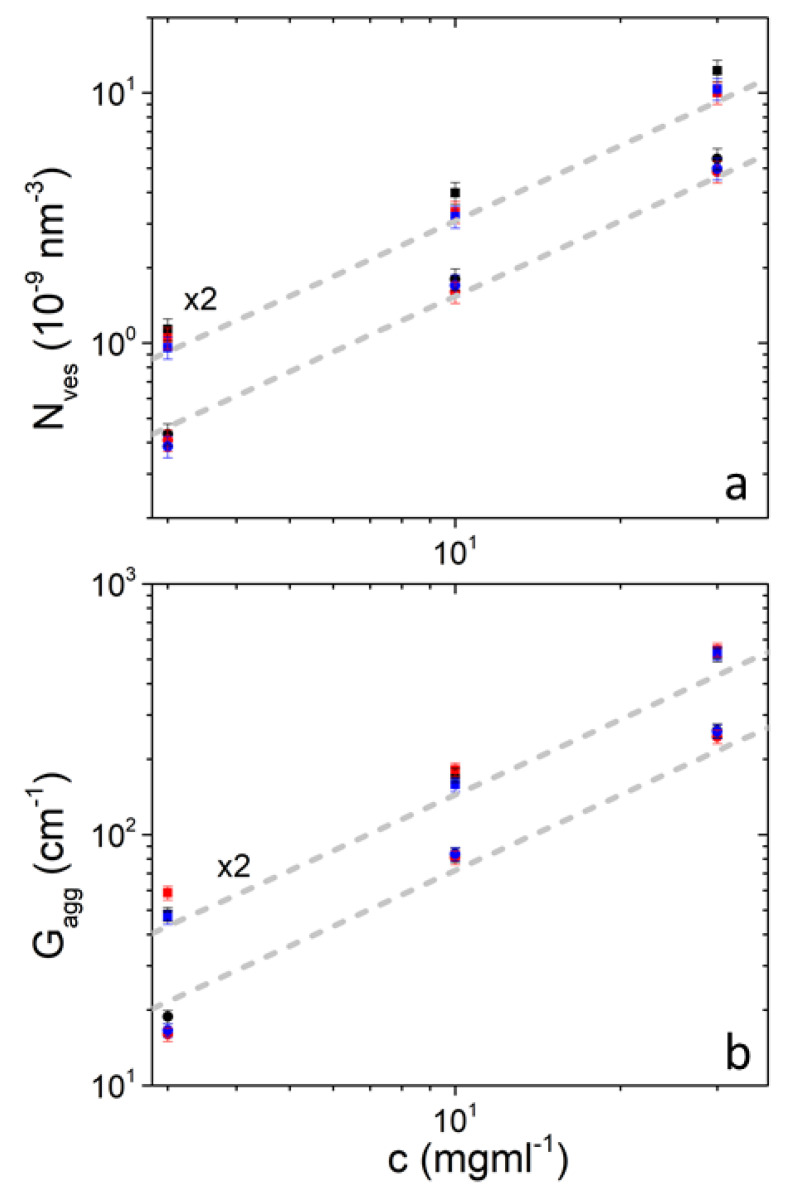
Number of vesicles per unit volume (**a**) and aggregates’ forward scattering (**b**) in solutions of DPPC/PEO-b-PCL1 (circles) and DPPC/PEO-b-PCL2 (squares) at 25 °C (black), 37 °C (red) and 45 °C (blue). Data from DPPC/PEO-b-PCL2 are multiplied by 2 for clarity. Gray dashed lines have slope equal to 1 and differ by a factor of 2.

The number of vesicles per unit volume $N_{ves}$ is proportional to concentration and therefore upon dilution the vesicles appear to significantly maintain their original structure (Figure 2). Similar is the case for aggregates and $G_{agg}$. It is also evident that temperature does not change these quantities significantly. $N_{ves}$ and $G_{agg}$ seem to be slightly higher in DPPC/PEO-b-PCL2 in comparison to DPPC/PEO-b-PCL1 (judging by their difference from the corresponding dashed lines). This is because of the increased hydrophobic content of PEO-b-PCL2 which favors stronger binding of the block copolymer to the vesicular bilayer. The interaction of PEO-b-PCL with DPPC liposomes has been confirmed by DSC studies [48].

The PDI is relatively high, which suggests that there is a broad distribution of vesicular sizes (Table 1). Approximately 95% percent of the vesicles have diameters between 10 and 70 nm. This is consistent with previous studies with cryo-TEM [48]. Polydispersities of up to 0.57 have been reported for dioleoylphosphatidylcholine (DOPC)-dioleoylphosphatidylethanolamine (DOPE) vesicles by Komorowski et al. [49]. In our previous work we found PDI about 0.30 for uni-lamellar DPPC vesicles using a symmetric distribution (Gaussian) and Kučerka al. reported similar PDI using Schulz distribution [45]. Schulz distribution is convenient for the creation of highly positively skewed distributions and has also been established in early fundamental studies on vesicles [50]. In a recent work of Huang et al., calculations of vesicular size distributions incorporated nonlinear elasticity and highly asymmetric (positively skewed) size distributions were predicted for bending stiffnesses in the order of 5–10 k_B_T [51]. The vesicle distributions showed a sharp rise at sizes higher than a minimum critical size which is followed by a long tail at higher sizes. Increasing bending stiffness distribution maximum shifted to higher sizes and to more symmetric shapes. This is similar to the behavior observed here as a function of increasing temperature. Remarkably, PDI is found to drop as temperature increases in both polymers which in combination with the size increase can be thought of as an increase of bending stiffness (Table 1). Therefore, incorporation of PEO-b-PCL diblock copolymer chains enhances the rigidity of the bilayers and at the same time it increases their mean diameter.

The mass concentration of vesicles in terms of mass of DPPC can be calculated as shown in the Supplementary Material. It is obvious that the mass of DPPC initially used for sample preparation is not finally incorporated in the mixed liposomes. The SLD of the copolymer blocks is between the values of the head and tail region and therefore incorporation into the vesicles could occur without detectable changes in the SLD profile. It is found that the mass incorporated in the mixed liposomes gradually increases for both copolymers (Table 2). This could be a sign of addition of new polymer on the vesicles as hydrophobic interaction is more intense. In PEO-b-PCL1 at 3 mg mL^−1^ a drop is observed from 37 to 45 °C that does not agree with the rest of the results. It could be due to some partial precipitation in this particular sample. Mass percentages in PEO-b-PCL2 are higher than the ones of PEO-b-PCL signifying the higher hydrophobicity of this copolymer.

The radius of gyration of the aggregates $R_{g,agg}$ is at 18 nm and their fractal exponent $D_{agg}$ is about 3 which identifies dense mass fractals or rough surface fractals [52,53]. The radius of gyration of the aggregates $R_{g,agg}$ shows a weak increase by temperature from about 17 to about 19 nm for both polymers. Fractal dimension $D_{agg}$ shows a weak increase which is actually negligible within experimental uncertainty. These values are found also not dependent on temperature. Cut-off length $R_{cut}$ is of the order 0.35$R_{g}$ in all cases and has a relatively high uncertainty.

**Figure 3:**
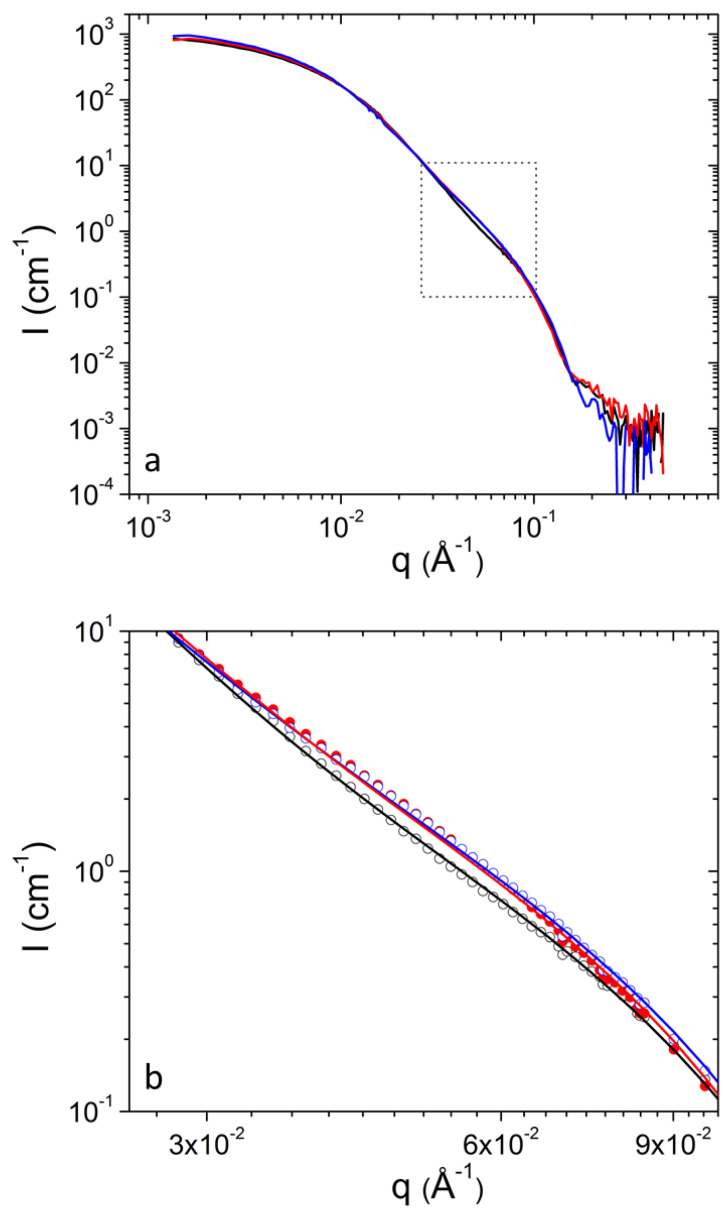
(**a**) SANS profiles from DPPC/PEO-b-PCL1 at 30 mg mL^−1^ at 25 °C (black), 37 °C (red) and 45 °C (blue). (**b**) Magnification of the region in rectangular from (**a**). Fitting lines are shown with the same color as the corresponding experimental data.

**Figure 4:**
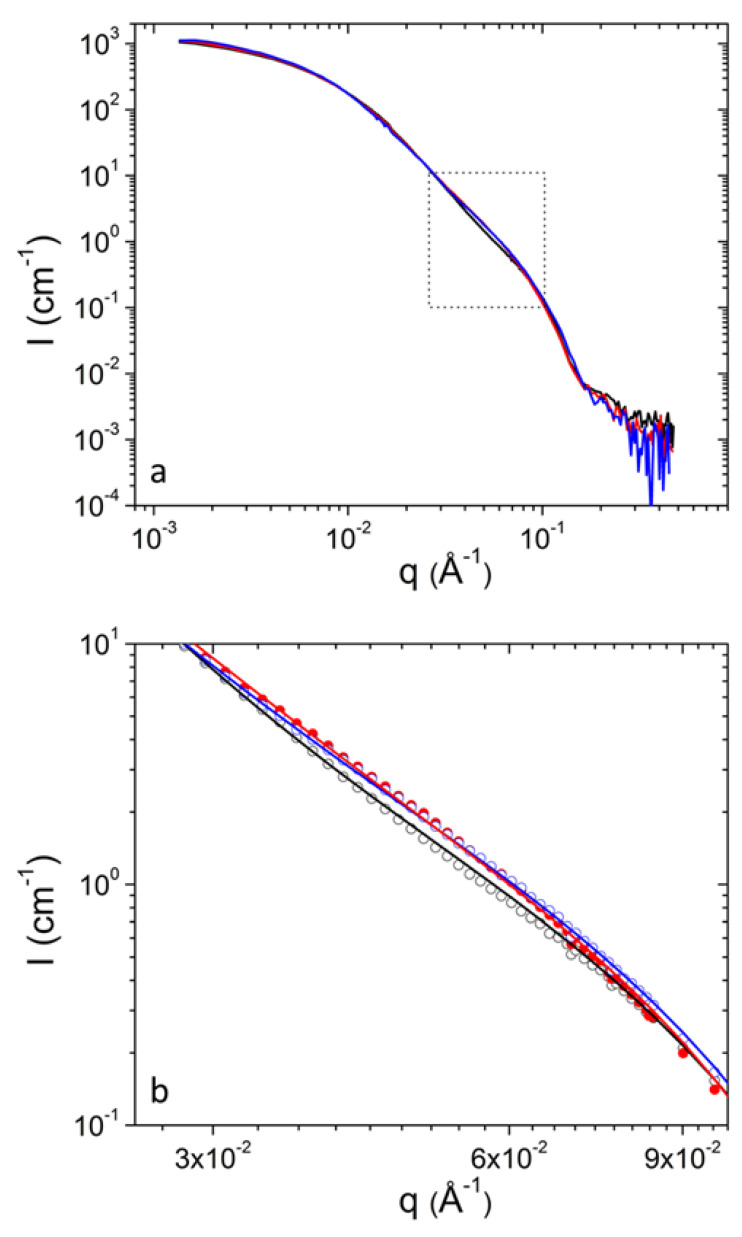
(**a**) SANS profiles from DPPC/PEO-b-PCL2 at 30 mg mL^−1^ at 25 °C (black), 37 °C (red) and 45 °C (blue). (**b**) Magnification of the region in rectangular from (**a**). Fitting lines are shown with the same color as the corresponding experimental data.

As it was discussed above, the parameters that were found to systematically increase as a function of temperature were the vesicular parameters R and D and the aggregates’ parameter $R_{g,agg}$. In order to confirm that the systematic changes in the extracted parameters as a function of temperature are reliable, we first plot the SANS profiles at 30 mg mL^−1^ at different temperatures (Figure 3 and Figure 4). Small, but clear change is found at 2.5 × 10^−2^−1.0 × 10^−1^ × Å^−1^. The uncertainty of the measured SANS intensity is smaller than the size of data points at this q range (not shown). Additionally, the fitting model follows the different data sets showing that fitting procedure distinguishes the small differences induced by temperature. In the q range from 2.5 × 10^−2^ to about 1 × 10^−1^ × Å^−1^ contributions from both aggregates and vesicles are significant. They are roughly equal ($I_{agg}\approx I_{ves}$) at about 2 × 10^−2^ × Å^−1^ and at *q* > 2 × 10^−2^ × Å^−1^ $I_{ves}$ gradually dominates scattered intensity. Any interdependency between parameters within or between the two form factors or any overlap of parameters within experimental uncertainty would not allow the extraction of conclusions regarding to which parameters really change by the temperature increase.

**Figure 5:**
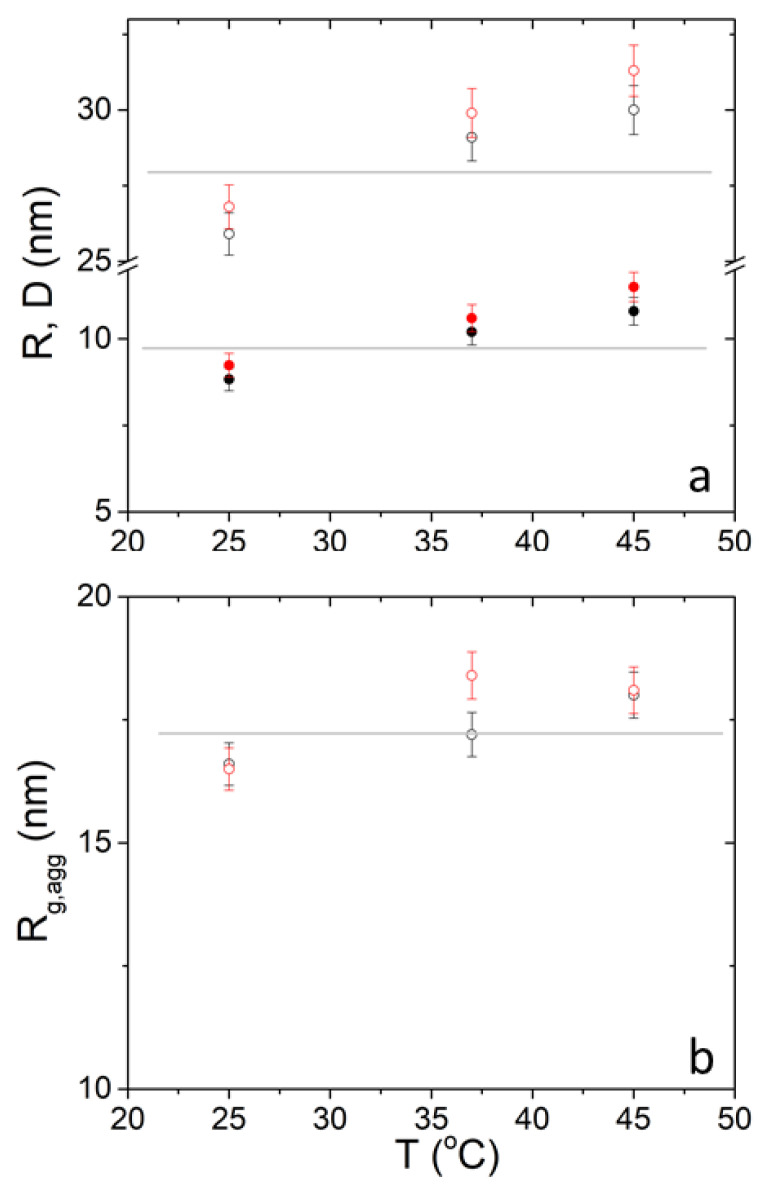
SANS-extracted R (closed circles) and D (open circles) (**a**) and $R_{g,agg}$ (**b**) from DPPC/PEO-b-PCL1 (black) and DPPC/PEO-b-PCL2 (red) at 30 mg mL^−1^. Horizontal lines are used as a guide to the eye.

The three parameters R, D and $R_{g,agg}$ for 30 mg mL^−1^ are plotted as a function of temperature for the sake of discussion in Figure 5. It is evident that there is a clear increase from 25 °C to 37 °C and an apparent increase from 37 °C to 45 °C which is however within the experimental error in the case of R and D. For $R_{g,agg}$ this is true only for PEO-b-PCL2 while for PEO-b-PCL1 it occurs from 37 °C to 45 °C. Actually, the significant change on the SANS profiles (Figure 2 and Figure 3b) are in the first temperature interval. In the second interval SANS profiles change only at the highest q values of the magnified region.

**Figure 6:**
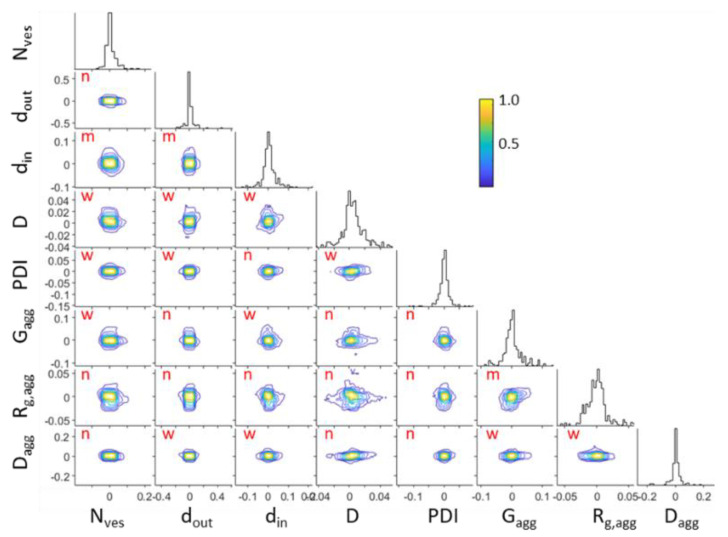
Corner plots of fitted parameters from DPPC/PEO-b-PCL1 at 30 mg mL^−1^ at 25 °C. Interdependencies are shown as “n” for negligible, “w” for weak and “m” for moderate.

Analyzing the Markov Chain Monte Carlo random walks in the parameter space after the convergence of the simulated annealing algorithm provides the mutual dependencies between the fitted parameters $P_{i}$ and their uncertainties $\delta P_{i}$. The corner plot of Figure 6 presents the distributions of the relative deviations of fitted parameters $P_{i}$ from their optimum values $P_{i}^{opt}$ i.e., $\frac{P_{i}-P_{i}^{opt}}{P_{i}^{opt}}$. The distributions of the individual parameters, from which the standard deviation can be extracted, are also shown. The probability distributions have been normalized to unity in regard to their maximum value (color bar of Figure 6). The correlation coefficients r of every pair of parameters have been calculated and the interdependency has been categorized [54] according to “negligible” for 0 ≤ |r| < 0.1,” weak” for 0.1 ≤ |r| < 0.4, “moderate” for 0.4 ≤ |r|<0.7, “strong” for 0.7 ≤ |r| < 0.9 and “very strong” for 0.9 ≤ |r|.

The probability distributions of pairs between the parameters of $I_{agg}$ i.e., $G_{agg}$, $R_{g,agg}$ and $D_{agg}$ and of $I_{ves}$ i.e., $N_{ves}$, $d_{out}$, $d_{in}$, D and PDI are only shown for clarity. In most cases correlations between parameters are negligible (17 occasions), many of them are weak (16 occasions) and in very few occasions they are moderate (3 occasions). There are no cases of strong or very strong correlation. Moderate correlation appears in the pairs $d_{in}-N_{ves}$, $d_{in}-d_{out}$ and $R_{g,agg}-G_{agg}$. Within these three pairs there is no combination of parameters between different scattering objects. This means that the separate contributions are very independent from each other. It can be safely assumed that the extracted parameters are reliably estimated and the multi-parameter model is justified.

**Figure 7:**
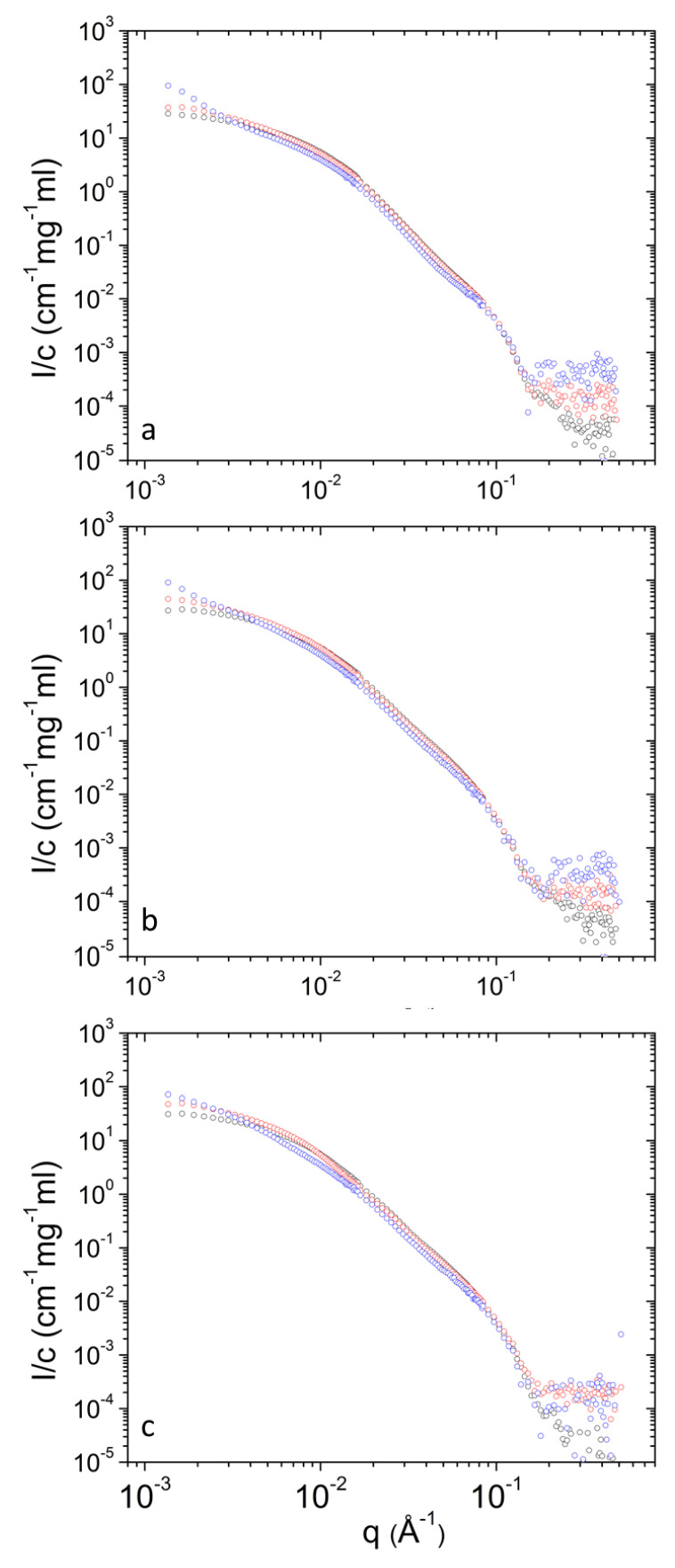
Concentration-normalized SANS profiles from DPPC/PEO-b-PCL1 at 3 (blue), 10 (red) and 30 (black) mg mL^−1^ at different temperatures i.e., (**a**) 25 °C, (**b**) 37 °C and (**c**) 45 °C.

**Figure 8:**
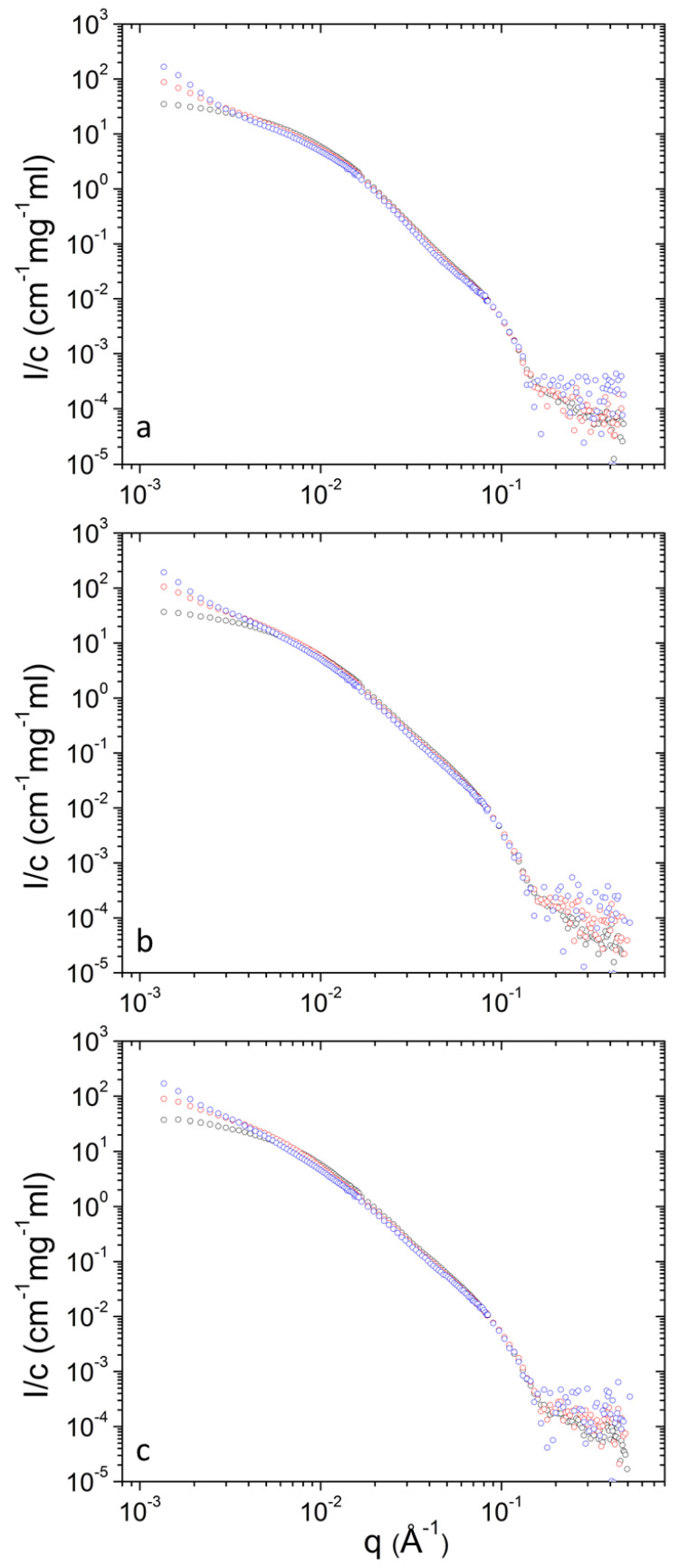
Concentration-normalized SANS profiles from DPPC/PEO-b-PCL2 at 3 (blue), 10 (red) and 30 (black) mg mL^−1^ at different temperatures i.e., (**a**) 25 °C, (**b**) 37 °C and (**c**) 45 °C.

Concentration-normalized SANS profiles are presented in Figure 7 and Figure 8. Non-trivial concentration dependence is found at low q. Specifically, the relative contribution from clusters $I_{0,clust}$ increases as concentration decreases (Table 1) upon dilution. This shows that upon dilution clustering of aggregates is more pronounced. The data of Table 2 shows that in most cases (except from 10 to 30 mg mL^−1^ at 45 °C for PEO-b-PCL1) the relative mass concentration of vesicles decreases as concentration decreases. This shows that there is an amount of material (either PEO-b-PCL or DPPC phospholipids or both) that leaves the vesicular state. Possibly this material is incorporated in clusters or causes stronger clustering of the initially formed aggregates.

## 4. Conclusions

In this work the structure of DPPC/PEO-b-PCL mixed liposomes has been investigated by SANS. Uni-lamellar vesicles were formed without the appearance of vesicles of higher lamellarity in contrast to pure DPPC and DPPC/MPOx vesicles of previous studies. Aggregates and clusters were also present in solution and were attributed to random associations of DPPC phospholipids and PEO-b-PCL block copolymer chains. The structure of the mixed liposomes was stable upon temperature increase and concentration changes. It was found that the length of the hydrophobic block of the copolymer affects the vesicles’ number and mass concentration as the PCL block interacts with the tail region of the bilayers. Enhancement of the hydrophobic interactions as temperature increases leads to size increase and polydispersity decrease in compatibility with current theoretical approaches on vesicles morphology. Statistical analysis supports the conclusions derived from the extracted parameters. This investigation shows that PEO-b-PCL can effectively stabilize well-defined uni-lamellar vesicles for delivery of proteins and drugs at the nanoscale.

## Figures and Tables

**Table 1:** SANS parameters extracted for DPPC/PEO-b-PCL1 solutions. Including number of vesicles per unit volume (N_ves_), vesicles’ diameter (D), vesicles’ polydispersity (PDI), forward scattering from aggregates (G_agg_), aggregates’ radius of gyration (R_g,agg_), aggregates’ fractal dimension (D_agg_), aggregates’ cut-off length (R_cut,agg_) and clusters’ scattering at the lowest q (I_0,clust_).

T (°C)	25			37			45			$\%\delta P_{i}/P_{i}\;*$
c (mg mL^−1^)	3	10	30	3	10	30	3	10	30	
DPPC/PEO-b-PCL1										
N_ves_ (10^−9^ nm^−3^)	0.432	1.80	5.45	0.409	1.60	4.84	0.386	1.70	4.98	10
d_out_ (nm)	0.61	0.64	0.60	0.59	0.62	0.58	0.62	0.63	0.60	29
d_in_ (nm)	2.86	3.16	2.88	3.03	2.93	3.13	2.74	2.94	2.91	9.6
R (nm)	8.66	8.66	8.83	9.95	9.98	10.2	10.5	10.8	10.8	3.7
D (nm)	25.6	25.6	25.9	28.5	28.5	29.1	29.4	29.9	30.0	2.7
PDI	0.853	0.853	0.827	0.760	0.760	0.730	0.705	0.696	0.695	5.9
G_agg_ (cm^−1^)	18.8	83.0	260	16.00	81.0	246	16.6	83.8	258	6.3
R_g.agg_ (nm)	16.4	16.8	16.6	17.5	17.5	17.2	18.5	18.5	18.0	2.6
D_agg_	2.93	2.91	2.94	2.95	2.94	2.77	3.07	3.09	2.95	26
R_cut.agg_ (nm)	7.61	6.60	6.52	6.86	6.86	6.68	6.58	6.58	6.33	19
I_0,clust_ (cm ^−1^)	231	164	162	210	171	241	159	236	127	8.0–30
DPPC/PEO-b-PCL2										
N (10^−9^nm^−3^)	0.567	1.99	6.14	0.523	1.67	5.00	0.479	1.60	5.18	10
d_out_ (nm)	0.64	0.60	0.62	0.61	0.59	0.64	0.60	0.62	0.58	29
d_in_ (nm)	2.87	2.96	2.86	3.15	3.08	2.89	2.94	2.97	2.91	9.6
R (nm)	8.28	8.72	9.24	9.87	10.7	10.6	11.7	11.8	11.5	3.7
D (nm)	24.8	25.7	26.8	28.4	29.9	29.9	31.7	32.0	31.3	2.7
PDI	0.900	0.838	0.790	0.729	0.700	0.700	0.649	0.643	0.663	5.9
G_agg_ (cm^−1^)	24.1	86.5	261	29.3	91.00	273	23.4	79.1	264	6.3
R_g.agg_ (nm)	16.8	16.7	16.5	19.3	18.4	18.4	18.8	18.7	18.1	2.6
D_agg_	2.85	2.98	2.94	3.30	3.14	3.14	3.19	3.17	3.07	26
R_cut.agg_ (nm)	6.87	6.67	6.58	6.04	5.88	5.88	6.29	6.25	6.20	19
I_0,clust_ (cm^−1^)	384	633	262	463	581	304	373	670	269	8.0–30 **

* estimation of uncertainties is described in the following discussion on Bayesian analysis. ** uncertainty is lowest at low concentrations where cluster contribution is significant.

**Table 2:** Percentage of mass concentration of vesicles relative to the nominal DPPC mass concentration (*c_ves_*/*c* · 100). The uncertainty *δc/c* on the presented concentrations is about 12%.

T (°C)	25			37			45		
**c** (**mg mL^−1^**)	**3**	**10**	**30**	**3**	**10**	**30**	**3**	**10**	**30**
DPPC/PEO-b-PCL1	23.8	29.7	30.5	29.0	33.8	35.0	28.4	39.3	38.3
DPPC/PEO-b-PCL2	29.4	32.9	37.0	35.0	38.4	38.3	41.7	42.6	44.0

## Supplementary Materials

The following are available online at https://www.mdpi.com/2073-4360/13/1/4/s1: 1. Contrast variation experiments and justification of SLD contrast extraction in absolute scale. 2. Derivation of vesicle mass concentration from SANS-extracted parameters.
