# Supplementary material for: Formation of Uni-Lamellar Vesicles in Mixtures of DPPC with PEO-b-PCL Amphiphilic Diblock Copolymers
Source: Polymers (Basel). 2020 Dec 22;13(1):4. doi: 10.3390/polym13010004 (PMC7792791; doi:10.3390/polym13010004)
Supplement: Supplementary file 1 [file polymers-13-00004-s001.pdf]

Formation of uni-lamellar vesicles in mixtures of DPPC with PEO-b-PCL amphiphilic diblock copolymers

Aristeidis Papagiannopoulos[*,1], Natassa Pippa[1,2], Costas Demetzos[2], Stergios Pispas[1] and Aurel Radulescu[3]

[1]Theoretical and Physical Chemistry Institute, National Hellenic Research Foundation, 48 Vassileos Constantinou Avenue, 11635 Athens, Greece.

[2]Department of Pharmaceutical Technology, Faculty of Pharmacy, Panepistimioupolis Zografou, National and Kapodistrian University of Athens, 15771 Athens, Greece.

[3]Jülich Centre for Neutron Science JCNS Forschungszentrum Jülich GmbH, Outstation at Heinz Maier-Leibnitz Zentrum (MLZ), Lichtenbergstraße 1, 85747 Garching, Germany.

Email: apapagiannopoulos@eie.gr

Supplementary Material

1. Contrast variation experiments and justification of SLD contrast extraction in absolute scale

In spherically symmetric objects the scattered intensity is $N \cdot \left|\int_0^\infty 4\pi r^2 \frac{sinqr}{qr} \Delta\rho(r) dr\right|^2$. Where $\Delta\rho(r) = \rho_{obj}(r) - \rho_w$ the radially averaged SLD of the scattering object. Another SLD profile $\Delta\rho'(r) = a \cdot \left(\rho_{obj}(r) - \rho_w\right)$ would give an identical scattering profile $I(q)$ provided that the number density of objects changed from N to N/a^2. Therefore, there is an infinite number of N and $\Delta\rho$ pairs that lead to identical $I(q)$.

One way to overcome this arbitrarity is to perform SANS experiments with contrast variation. In this case the experiments can be performed in a solvent with a different contrast assuming that the morphology of the scattering objects remains unaltered. The scattered intensity $I(q)$ from a profile $\Delta\rho(r) = \rho_{obj}(r) - \rho_w$ would change to another $I(q)$ that corresponds to the profile $\Delta\rho_{cv}(r) = \rho_{obj}(r) - \rho_w - \delta\rho_w$ where $\delta\rho_w$ is the change of water scattering length demsity caused by contrast variation. If the initial $\Delta\rho(r)$ changes to $a \cdot \Delta\rho(r)$ the initial fit would still be optimal by changing N to N/a^2. For the experiment with the mixed solvent however this change would lead to $\Delta\rho_{cv}'(r) = a \cdot \left(\rho_{obj}(r) - \rho_w\right) - \delta\rho_w$ and a simple normalization of N would not provide an optimal $I(q)$. We performed experiments in mixed D_2O/H_2O solvent (Fig. S1) with 50/50 and 33.3/66.7 v/v. The solvent SLD changes from its value in D_2O $\rho_{D2O} = 6.4 \cdot 10^{-6}$Å^{-2} to $\rho_w = 2.92 \cdot 10^{-6}$Å^{-2} and $\rho_w = 1.76 \cdot 10^{-6}$Å^{-2} respectively. The SLD profiles extracted from the fits (Table 1) were used to calculate the SANS data from contrast variation experiments (Fig. S1). All parameters were the same except from the SLD of the solvent. The resulting SANS data were optimal for all contrasts which proves that the SLD profiles and number densities were extracted in absolute scale.

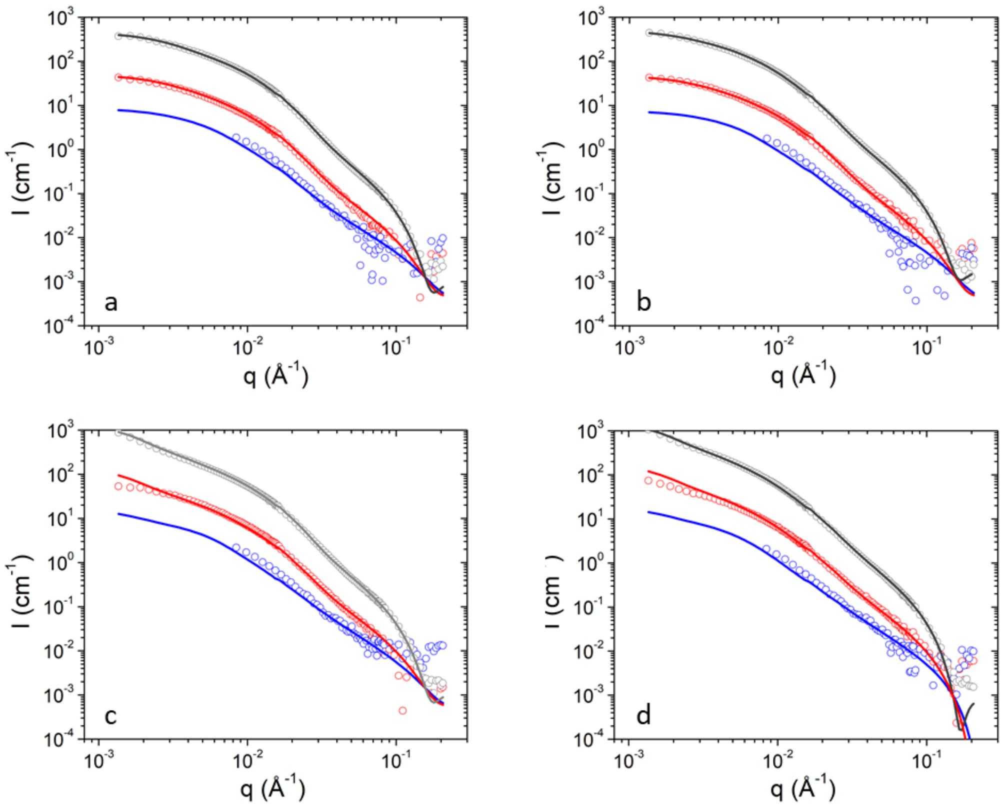

Fig S1. Contrast variation test on DPPC/PEO-b-PCL1 (a at 25 °C, and b at 37 °C) and DPPC/PEO-b-PCL2 (c at 25 °C, and d at 37 °C) at 10 mgml^{-1} in D_2O/H_2O mixed solvent with volume ratios 100/0 (gray), 50/50 (red) and 33.3/66.7 (blue) v/v. Gray lines are fits on the experimental data (as described in the text). Red and blue lines are calculated SANS profiles with the same parameters as in experiments in pure D_2O. We show the SANS data up to q=0.2 Å^{-1} because background subtraction at higher q was problematic. This was caused by the high contribution of incoherent scattering in the samples that contain H_2O.

The above arguments also hold for any geometry and SLD distribution within scattering objects. In order for the scattering form factor of aggregates and clusters to be reproducible their SLD was optimized at $\rho_{agg} = \rho_{clust} = 1.32 \cdot 10^{-6}$ Å^{-2}. In other words, contrast variation allowed the estimation of the volume-average SLD of these objects. Apparently, these objects may consist of PEO-b-PCL and DPPC as the SLD is within the values that correspond to these components.

2. Derivation of vesicle mass concentration from SANS-extracted parameters

The volume of a bilayer in a single vesicle is $V_{ves} = \frac{4\pi}{3}[(r^3 + 2d_{out} + d_{in})^3 - r^3]$. Using the volume that corresponds to a single lipid [A. Koutsioubas, J Phys Chem B, 120 (2016) 11474-11483] i.e. V_{lip}=1215.5 Å^3, their molar mass M_{lip}=734 gmol^{-1} and the number density of vesicles N_{ves}, the mass concentration of monodisperse vesicles of internal radius r in solution can be

extracted as $c_{ves}(r) = \frac{N_{ves} \cdot V_{ves}}{V_{lip}} M_{lip}/N_A$. For polydisperse vesicles with Schulz distribution $c_{ves} = \frac{\left(\frac{z+1}{R}\right)^{z+1}}{\Gamma(z+1)} \int_0^{+\infty} r^z \cdot exp\left(-\frac{z+1}{R} \cdot r\right) c_{ves}(r) dr$.

We have the calculated SLD of PEO and PCL blocks at $1.2 \cdot 10^{-6}$ Å^{-2} and $2.0 \cdot 10^{-6}$ Å^{-2} respectively. These values lie between the ones of the hydrophobic and hydrophilic components of the DPPC lipids.
